# Plant-produced SARS-CoV-2 receptor binding domain (RBD) variants showed differential binding efficiency with anti-spike specific monoclonal antibodies

**DOI:** 10.1371/journal.pone.0253574

**Published:** 2021-08-11

**Authors:** Kaewta Rattanapisit, Christine Joy I. Bulaon, Narach Khorattanakulchai, Balamurugan Shanmugaraj, Kittikhun Wangkanont, Waranyoo Phoolcharoen

**Affiliations:** 1 Baiya Phytopharm Co., Ltd., Bangkok, Thailand; 2 Research Unit for Plant-produced Pharmaceuticals, Chulalongkorn University, Bangkok, Thailand; 3 Department of Pharmacognosy and Pharmaceutical Botany, Faculty of Pharmaceutical Sciences, Chulalongkorn University, Bangkok, Thailand; 4 Center of Excellence for Molecular Biology and Genomics of Shrimp (GCE 6302823006-1), Department of Biochemistry, Faculty of Science, Chulalongkorn University, Bangkok, Thailand; 5 Molecular Crop Research Unit (GRU 6407023008-1), Department of Biochemistry, Faculty of Science, Chulalongkorn University, Bangkok, Thailand; 6 Department of Biochemistry, Faculty of Science, Chulalongkorn University, Bangkok, Thailand; Waseda University: Waseda Daigaku, JAPAN

## Abstract

Severe acute respiratory syndrome coronavirus-2 (SARS-CoV-2) is responsible for the ongoing coronavirus disease (COVID-19) pandemic which is characterized by respiratory illness and severe pneumonia, and currently accounts for > 2.5 million deaths worldwide. Recently, diverse mutations in the spike protein of SARS-CoV-2 were reported in United Kingdom (Alpha) and South Africa (Beta) strains which raise concerns over the potential increase in binding affinity towards the host cell receptor and diminished host neutralization capabilities. In order to study the effect of mutation in the binding efficiency of SARS-CoV-2 receptor binding domain (RBD) with anti-SARS-CoV/CoV-2 monoclonal antibodies (mAbs), we have produced SARS-CoV-2 RBD and two variants SARS-CoV-2 RBD (Alpha RBD and Beta RBD) in *Nicotiana benthamiana* by transient expression. Plant-produced SARS-CoV-2 RBD-Fc, Alpha RBD-Fc and Beta RBD-Fc exhibited specific binding to human angiotensin converting enzyme 2 (ACE2) receptor determined by ELISA. Intriguingly, the binding of plant-produced SARS-CoV-2 RBD proteins to plant-produced mAbs CR3022, B38, and H4 was found to be different depending on the variant mutation. In contrary to the plant-produced SARS-CoV-2 RBD-Fc and Alpha RBD-Fc, Beta RBD-Fc variant showed weak binding affinity towards the mAbs. The result suggested that the Beta RBD variant might have acquired partial resistance to neutralizing antibodies compared to other variants. However, further studies with sera from convalescent or vaccinated individuals are required to confirm this finding.

## Introduction

The emergence of the novel pathogenic coronavirus designated as severe acute respiratory syndrome coronavirus 2 (SARS-CoV-2) is responsible for the ongoing coronavirus disease 2019 (COVID-19) outbreak. The virus was reported to be originated from animals and subsequently transmitted to humans. The virus has reached several countries with more than 115 million of SARS-CoV-2 infected cases and over 2.5 million deaths around the world [1]. The overwhelming number of cases have massively affected the global economy, disrupted international relations, and continuously raised threats to global health and safety. In addition, recent challenges on public health and social measures have garnered concerns regarding the occurrence of multiple SARS-CoV-2 variants [2] as these mutations might increase viral infectivity, disease severity, and even alter efficiency of available vaccine, antibody therapies, and other preventive countermeasures.

Genetic variations from the wildtype coronavirus SARS-CoV-2 are inevitable and naturally emerge over the time. At present, new virus variants of SARS-CoV-2 have been documented and have since been circulating across countries amid the pandemic. The emergence of two novel virus lineages, B.1.1.7 [3] and B.1.351 [4], were initially identified in United Kingdom (Alpha) and South Africa (Beta) [5]. These variants of concern (VOC) contain genomic variability that may jeopardize the existing preventive countermeasures. In late 2020, a SARS-CoV-2 variant, VOC 202012/01 (lineage B.1.1.7) predominantly circulated in the south of UK [6]. The variant carries large number of mutations from the parental virus with eight amino acid substitutions in the spike (S) protein. Notably, the mutations at key sites in the receptor-binding domain (RBD) of S glycoprotein include N501Y, A570D, and D614G [7]. Later 501Y.V2 variant (lineage B.1.351) emerged independently in South Africa [8] and has since spread widely in countries outside African continents. It shares some similar spike mutations with the Alpha variant and also contains multiple mutations at important residues in the RBD including K417N and E484K [9]. Preliminary evidences suggest that these mutations in the SARS-CoV-2 RBD can potentially impact virulence, transmissibility, and sensitive to neutralizing antibodies [10–12].

Ever since the pandemic, vaccine development has progressed around the globe and few of the vaccines are currently deployed to control the disease. The new viral variants usually evolve to evade the available vaccines. However, prior investigations suggest that response of SARS-CoV-2 variants against vaccine-induced immunity vary among individuals. For instance, efficacy of antibody neutralization against circulating variants carrying multiple mutations demonstrated slight reduction in the vaccine-elicited neutralizing antibody titers or no loss of antibody response [13], which was contrary to the lower levels of neutralizing antibodies accumulated *in vitro* [14] or complete resistance to neutralization [15] from a COVID-19 convalescent plasma. Hence, possibilities of SARS-CoV-2 variants to escape immunity induced by vaccines should be critically evaluated.

The rationale of this study is to determine the effect of mutations in the RBD of SARS-CoV-2 on the binding affinity with anti-SARS-CoV and anti-SARS-CoV-2 monoclonal antibodies (mAbs) for preliminary test without the need of virus safety control. Hence, the RBD of SARS-CoV-2 and variants Alpha and Beta were produced in *Nicotiana benthamiana* and its binding affinity with host cell receptor, angiotensin-converting enzyme 2 (ACE2), was investigated by ELISA. Furthermore, the binding of RBD variants with three plant-produced mAbs CR3022, B38, and H4 were evaluated.

## Materials and methods

### Construction of SARS-CoV-2 RBD-Fc, Alpha and Beta variants RBD-Fc in geminiviral vector for transient expression

**Fig 1:**
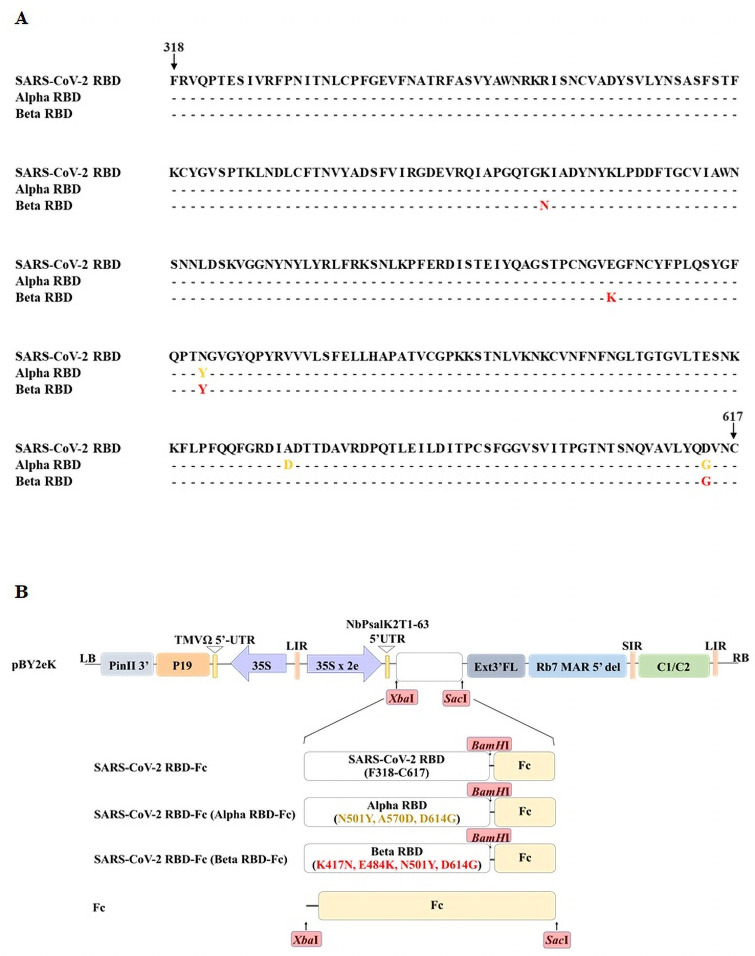
Amino acid sequence alignment of SARS-CoV-2, Alpha, and Beta RBDs and schematic representation of gene constructs in pBY2eK. (A) Amino acid alignment of SARS-CoV-2 RBD, Alpha RBD, and Beta RBD. Yellow and red color indicates amino acid mutation in Alpha RBD and Beta RBD, respectively. (B) Schematic representation of pBY2eK vector. T-DNA regions of the pBY2eK vector consists of P35S (cauliflower mosaic virus (CaMV) 35S promoter), NbPsaK2T 5’UTR (5′ untranslated region), Ext3′FL (3′ full length of the tobacco (Nicotiana tabacum) extension gene), Rb7 MAR 5’ del (tobacco RB7 promoter), SIR (short intergenic region of BeYDV genome), LIR (long intergenic region of BeYDV genome), C2/C1 (bean yellow dwarf virus (BeYDV) open reading frames C1 and C2 encoding for replication initiation protein (Rep) and RepA), P19 (P19 gene from tomato bushy stunt virus (TBSV)),TMVΩ 5’-UTR (5’ untranslated region of tobacco mosaic virus Ω), and PinII 3′ (terminator from potato proteinase inhibitor II gene). The SARS-CoV-2 RBD-Fc or Alpha RBD-Fc or Beta RBD-Fc or Fc gene was inserted into the pBY2eK (*Xba*I and *Sac*I enzyme sites).

The construct of SARS-CoV-2 RBD-Fc was produced from the previous study [16]. In addition, the amino acid sequences (F318 to C617) of Alpha and Beta variants RBD (GenBank accession number: QQX0 1934.1 (Alpha) and QSH75306.1 (Beta)) (Fig 1A) were retrieved. The nucleotide sequences encoding Alpha and Beta variants RBD flanked with the murine leader sequence [17] and 1xGGGGS were codon-optimized for *Nicotiana benthamiana* expression and commercially synthesized (Genewiz, Suzhou, China). The Fc fragment linked with an ER retention signal SEKDEL motif [16] was amplified by polymerase chain reaction (PCR) using BamHI-2xGGGGS-Fc forward primer and SacI-SEKDEL-Fc reverse primer as listed on Table 1. Each variant SARS-CoV-2 RBD was digested with *Xba*I and *BamH*I restriction enzymes (New England Biolabs, Hitchin, UK) and similarly Fc fragment was digested with *BamH*I and *Sac*I restriction enzymes (New England Biolabs, Hitchin, UK). The digested fragments were gel-purified and both fragments were ligated into geminiviral vector pBYR2eK2Md (pBY2eK) [18] using T4 DNA ligase (New England Biolabs, Hitchin, UK). Meanwhile, the nucleotide sequence encoding Fc protein (Fc) was amplified by PCR using XbaI-3xGGGGS-Fc forward primer and SacI-SEKDEL-Fc reverse primer provided in the Table 1. The resulting PCR product was gel-purified and ligated into pBY2eK expression vector with *Xba*I and *Sac*I restriction enzymes. The developed pBY2eK-RBD-Fc fusion protein constructs containing either SARS-CoV-2 RBD-Fc, Alpha RBD-Fc or Beta RBD-Fc and the pBY2eK-Fc (Fig 1B) were transformed into *Escherichia coli* strain DH10B competent cells by heat shock method and the recombinant plasmids were confirmed by restriction enzyme (*Xba*I and *Sac*I) digestion and DNA sequencing. The confirmed plasmids were transformed to *Agrobacterium tumefaciens* GV3101 *via*., electroporation.

**Table 1:** List of primers used in this study.

Primer Name	Sequence (5’-3’)
BamHI-2xGGGGS-**Fc**	GGATCCGGAGGTGGAGGTTCTGGAGGTGGAGGTTCA**CCACCATGTCCAGCTCCAG**
SacI-SEKDEL-**Fc**	GAGCTCTTAAAGCTCATCCTTCTCAGA**CTTGCCAGGGGACAAAGAAAGG**
XbaI-3xGGGGS-**Fc**	TCTAGAATGGGAGGTGGAGGTTCTGGAGGTGGAGGTTCTGGAGGTGGAGGTTCA**CCACCATGTCCAGCTCCAG**

### Transient expression of SARS-CoV-2 RBD-Fc, Alpha and Beta variants RBD-Fc in *N*. *benthamiana*

Wild type *N*. *benthamiana* plants were grown in a controlled room with 16 h light/ 8 h dark cycle at 25–28°C for 6–8 weeks. *Agrobacterium* cells containing either the pBY2eK-SARS-CoV-2 RBD-Fc or Alpha RBD-Fc or Beta RBD-Fc construct was cultured overnight at 28°C with continuous shaking at 200 rpm. Overnight bacterial cultures were centrifuged at 4,000 *g* for 10 min at room temperature and the pellets were resuspended in infiltration buffer (10mM 2-[N-morpholino] etanesulfonic acid (MES), 10mM MgSO_4_, at pH 5.5) to get a final OD_600_ of 0.4. Each cell suspension containing either pBY2eK-SARS-CoV-2 RBD-Fc or any of the variants RBD-Fc was agroinfiltrated in *N*. *benthamiana* plants by syringe infiltration. The infiltrated plants were incubated in a controlled room for 3 days at 28°C. The concentration of the crude protein extracts was calculated in comparison with bovine serum albumin (BSA) standard (Thermo Scientific, Illinois, USA) by the Bradford method (Bio-rad, California, USA).

### Recombinant protein expression and purification

The cultured *Agrobacterium* cells containing the pBY2eK-SARS-CoV-2 RBD-Fc or Alpha RBD-Fc or Beta RBD-Fc or Fc construct was diluted in infiltration buffer to an OD_600_ of 0.2 for vacuum infiltration into *N*. *benthamiana*. Infiltrated plants were harvested 3 days post infiltration and extracted with 1xPBS extraction buffer (phosphate-buffered saline: 137mM NaCl, 2.7mM KCl, 4.3mM Na_2_HPO_4_, 1.47mM KH_2_PO_4_, at pH 7.4). The homogenized plant crude extracts were centrifuged at 26,000 *g* for 40 min at 4°C. Each supernatant was filtered with 0.45 μm membrane filter and loaded into protein A bead (GE healthcare, Illinois, USA) column. The column was further washed three times with 1xPBS and eluted with 100mM glycine at pH 2.7. The purified protein was neutralized with 1.5M Tris-HCl at pH 8.8. The concentration of purified plant-produced proteins was measured in comparison with the BSA and the internal plant-produced SARS-CoV-2 RBD-Fc standard by the Bradford assay.

### Sodium dodecyl sulfate polyacrylamide gel electrophoresis (SDS-PAGE) and western blot

The plant crude extracts or purified plant-produced proteins were further analyzed by SDS-PAGE and western blot. For plant crude extracts, proteins were separated by 4–15% SDS-PAGE gel under reducing and non-reducing conditions using wild type *N*. *benthamiana* extract as the negative control. Similarly, purified plant-produced proteins were subjected to 4–15% SDS-PAGE specifically SARS-CoV-2 RBD-Fc, Alpha RBD-Fc and Beta RBD-Fc was analyzed under both reducing and non-reducing condition whereas Fc protein was separated under non-reducing condition. The separated proteins were stained with InstantBlue^®^ (Abcam, Cambridge, UK) for visualization or transferred to nitrocellulose membrane (Bio-rad, California, USA) for western blotting. The membranes were blocked with 5% skim milk and probed either with horseradish peroxidase (HRP)-conjugated goat anti-human IgG (Southern Biotech, Alabama, USA) diluted 1:10,000 in 3% skim milk or with rabbit anti-SARS-CoV-2 spike protein (RBD) mAb (Invitrogen, California, USA) diluted 1:5,000 and HRP-conjugated goat anti-rabbit IgG (Boster Bio, California, USA) diluted 1:5,000 in 3% skim milk. The membranes were washed and developed by chemiluminescence using ECL detection reagent (Promega, Wisconsin, USA) as per manufacturer’s instructions.

### Binding of plant-produced SARS-CoV-2 RBD-Fc, Alpha and Beta variants RBD-Fc to ACE2 receptor by ELISA

A 96-well ELISA plate (Corning, New York, USA) was coated with 25 μl of 2 μg/ml human ACE2 protein (Sino Biological, Beijing, China) and incubated overnight at 4°C. The coated plate was washed three times with 1xPBST (1xPBS with 0.05% Tween20) and blocked with 5% skim milk for 1 h at 37°C. Then, the plate was washed three times with 1xPBST and incubated with 0.02–5.00 μg/ml of purified plant-produced SARS-CoV-2 RBD-Fc or Alpha RBD-Fc or Beta RBD-Fc or Fc protein for 2 h at 37°C. The plate was washed three times with 1xPBST and incubated with HRP-conjugated goat anti-human IgG diluted 1:2,000 in 1xPBS for 1 h at 37°C. The plate was washed and the signal was detected with 25 μl of TMB substrate (Promega, Wisconsin, USA) and stopped with 25 μl of 1M H_2_SO_4_. The absorbance was measured at 450 nm using SpectraMax M5 (Molecular devices, California, USA). The experiment was performed in three replicates and the data are presented as mean±SD.

### Binding of plant-produced CR3022, B38 and H4 mAbs to plant-produced SARS-CoV-2 RBD-Fc, Alpha and Beta variants RBD-Fc by ELISA

Briefly, 25 μl of 2 μg/ml purified plant-produced SARS-CoV-2 RBD-Fc or Alpha RBD-Fc or Beta RBD-Fc or Fc protein was coated on a 96-well ELISA plate and incubated overnight at 4°C. The coated plate was washed with 1xPBST and blocked with 5% skim milk for 1 h at 37°C. The plate was washed with 1xPBST and incubated either with 0.31–40.00 μg/ml of purified plant-produced mAbs CR3022 or B38 or H4 [17, 19] for 2 h at 37°C. Then, the plate was washed with 1xPBST and incubated with HRP-conjugated goat anti-human Kappa (Southern Biotech, Alabama, USA) diluted 1:2,000 in 1xPBS for 1 h at 37°C. The plate was washed and the signal was detected with 25 μl of TMB substrate and stopped with 25 μl of 1 M H_2_SO_4_. The absorbance was measured at 450 nm using SpectraMax M5. The experiment was performed in three replicates and the data are presented as mean±SD.

### Structural analysis of SARS-CoV-2 RBD-Fc proteins binding to mAbs

Crystal structures of SARS-CoV-2 RBD in complex with the Fab fragments of the CR3022 mAb (PDB ID 6W41) [20] and B38 mAb (PDB ID 7BZ5) [21] were used for structural analysis. The RBD portion of the structures were aligned together using PyMOL (PyMOL Molecular Graphics System, Version 2.5.0a0 Schrödinger, LLC) (Cα RMSD = 0.518 Å). *In silico* mutagenesis were performed and the structure figures were generated using PyMOL.

## Results

### Expression of SARS-CoV-2 RBD-Fc, Alpha and Beta variants RBD-Fc in *N*. *benthamiana*

**Fig 2:**
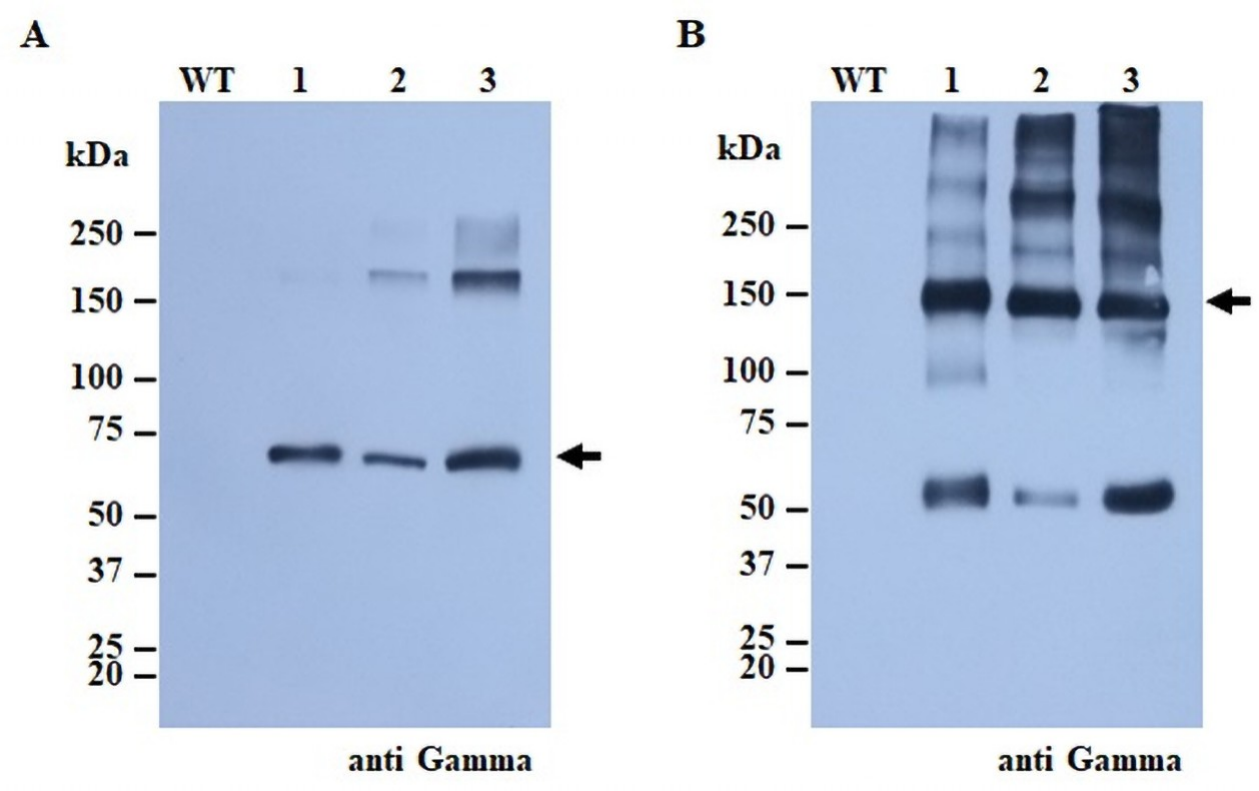
Transient expression of SARS-CoV-2 RBD-Fc and variants in *N*. *benthamiana*. Agroinfiltrated *N*. *benthamiana* leaves were extracted and protein expression was assessed by western blotting using HRP-conjugated goat anti-human IgG Fc specific antibody under reducing condition (A) and non-reducing condition (B). Lane WT: crude extract of wild type *N*. *benthamiana*; Lane 1: crude extract of *N*. *benthamiana* agroinfiltrated with pBY2eK-SARS-CoV-2 RBD-Fc; Lane 2: crude extract of *N*. *benthamiana* agroinfiltrated with pBY2eK-Beta RBD-Fc; Lane 3: Lane 1: crude extract of *N*. *benthamiana* agroinfiltrated with pBY2eK-Alpha RBD-Fc. The arrow indicated the expected band. The experiment was performed in three times repeat.

The nucleotide sequence of SARS-CoV-2 RBD and variant RBD, Alpha and Beta, strains (Fig 1A) were codon-optimized and fused along with Fc fragment and subsequently cloned with plant expression geminiviral vector pBY2eK (Fig 1B). The recombinant vectors harbouring the SARS-CoV-2 RBD-Fc, Alpha RBD-Fc, Beta RBD-Fc, and Fc were propagated and transformed into *Agrobacterium tumefaciens*. *Agrobacterium* containing either of the constructs were infiltrated into *N*. *benthamiana*. Three days after infiltration, the infiltrated leaves were harvested and the expression of SARS-CoV-2 RBD-Fc, Alpha RBD-Fc, and Beta RBD-Fc was detected by western blot using HRP-conjugated anti-human IgG (Fig 2). The expected protein band at approximately 75 kDa was observed under reducing conditions (Fig 2A) and 150 kDa under non-reducing conditions (Fig 2B).

### Purification of recombinant protein from *N*. *benthamiana* crude extracts

**Fig 3:**
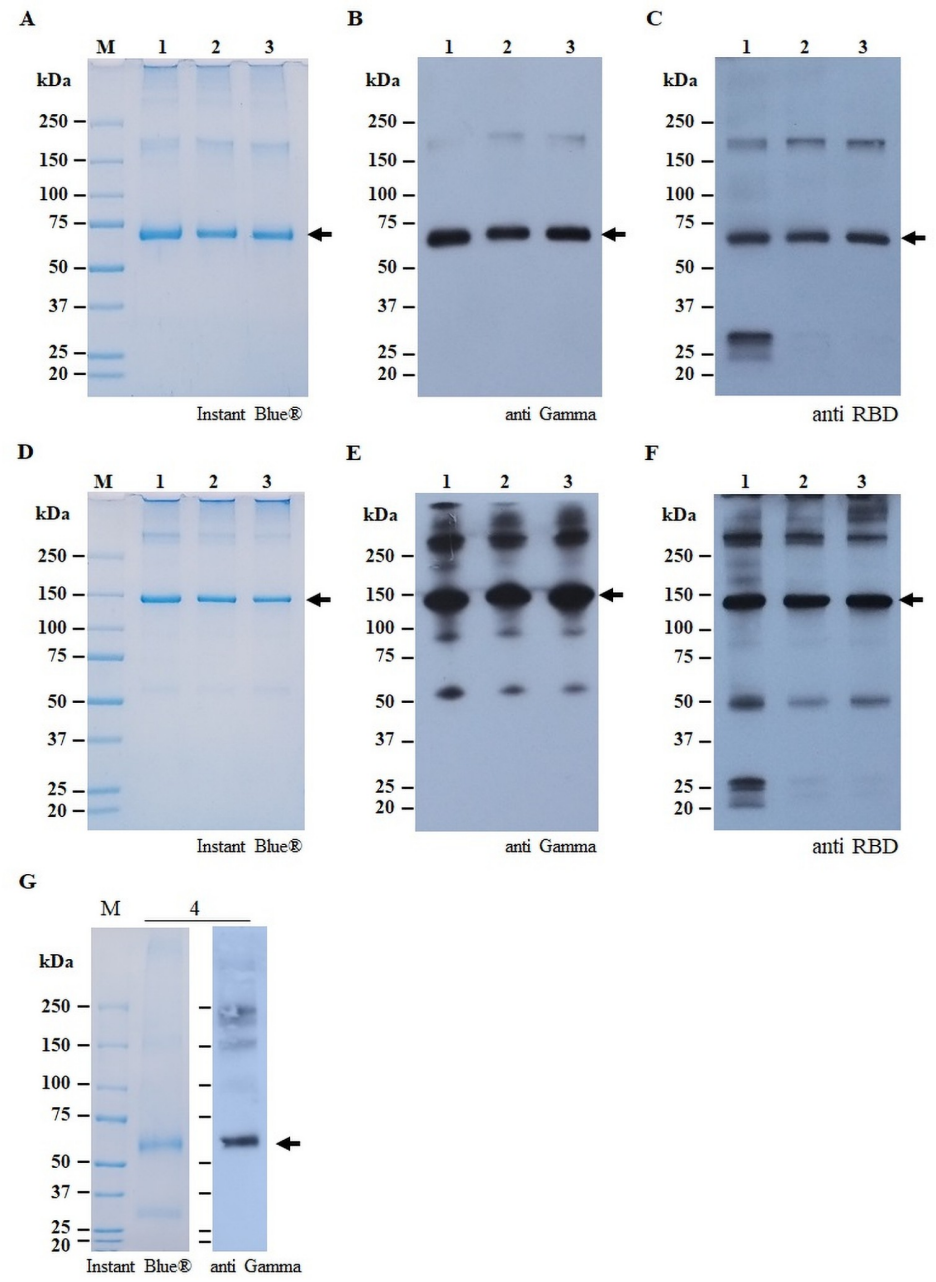
SDS-PAGE and western blot analysis of purified plant-produced SARS-CoV-2 RBD-Fc, variants, and Fc. Purified plant-produced SARS-CoV-2 RBD-Fc, Alpha RBD-Fc, and Beta RBD-Fc variants were evaluated by SDS-PAGE and western blot under reducing condition (A, B, and C) and non-reducing condition (D, E, and F). In addition, purified plant-produced Fc was assessed by SDS-PAGE and western blot under nonreducing condition (G). The proteins were separated by 4–15% SDS PAGE and stained with InstantBlue^®^ (A and D). For western blot, the proteins were transferred onto nitrocellulose membrane and probed with HRP-conjugated goat anti-human IgG (B and E) or rabbit anti-SARS-CoV-2 spike protein (RBD) mAb (C and F). Lane 1: purified plant-produced SARS-CoV-2 RBD-Fc; Lane 2: purified plant-produced Beta RBD-Fc protein; Lane 3: purified plant-produced Alpha RBD-Fc protein; Lane 4: purified plant-produced Fc protein. The arrow indicated the expected band.

Protein-A affinity chromatography was used to purify the SARS-CoV-2 RBD-Fc, Alpha RBD-Fc and Beta RBD-Fc and Fc protein from *N*. *benthamiana* leaves. The purified plant-produced proteins were subjected to SDS-PAGE and western blot analysis (Fig 3). The expected band at 75 kDa was observed in the SDS-PAGE stained gels under reducing condition (Fig 3A) and 150 kDa under non-reducing condition which corresponds to RBD-Fc protein (Fig 3D). In western blot, the plant-produced RBD-Fc proteins were detected at 75 and 150 kDa under reducing and non-reducing condition by using HRP-conjugated anti-human IgG (Fig 3B and 3E) and anti-SARS-CoV-2 spike protein (RBD) mAb (Fig 3C and 3F). The average yield of RBD-Fc produced in *N*. *benthamiana* was observed to be 20 μg/g leaf fresh weight.

### Binding of plant-produced SARS-CoV-2 RBD-Fc, Alpha and Beta variants RBD-Fc to ACE2 receptor

**Fig 4:**
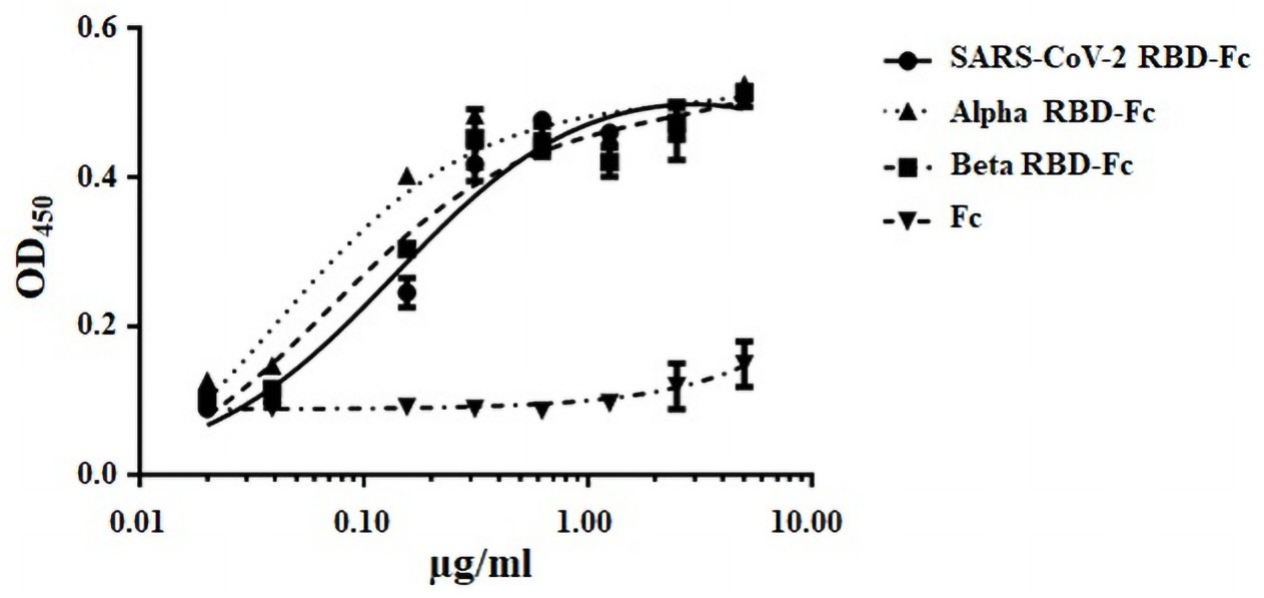
*In vitro* binding of purified plant-produced SARS-CoV-2 RBD-Fc and variants to human ACE2. The binding affinity to ACE2 was determined by ELISA. Two-fold dilution of plant-produced SARS-CoV-2 RBD-Fc, Alpha RBD-Fc, Beta RBD-Fc, and Fc proteins were incubated on plates coated with human ACE2 protein. The bound protein was detected with HRP-conjugated goat anti-human IgG. The data are the mean±SD of triplicates assay from each concentration.

SARS-CoV-2 RBD was reported to interact with human ACE2 receptor for host cell entry [22]. The binding efficiency of the plant-produced SARS-CoV-2 RBD-Fc protein, Alpha RBD-Fc, and Beta RBD-Fc to ACE2 were determined by ELISA. The plant-produced Fc protein was used as a negative control. The results showed that plant-produced SARS-CoV-2 RBD-Fc and variants RBD-Fc exhibit similar binding affinity with the ACE2 protein, compared to the control plant-produced Fc protein (Fig 4).

### Binding of SARS-CoV-2 RBD-Fc, Alpha and Beta variants RBD-Fc to plant-produced mAbs CR3022, B38, and H4

**Fig 5:**
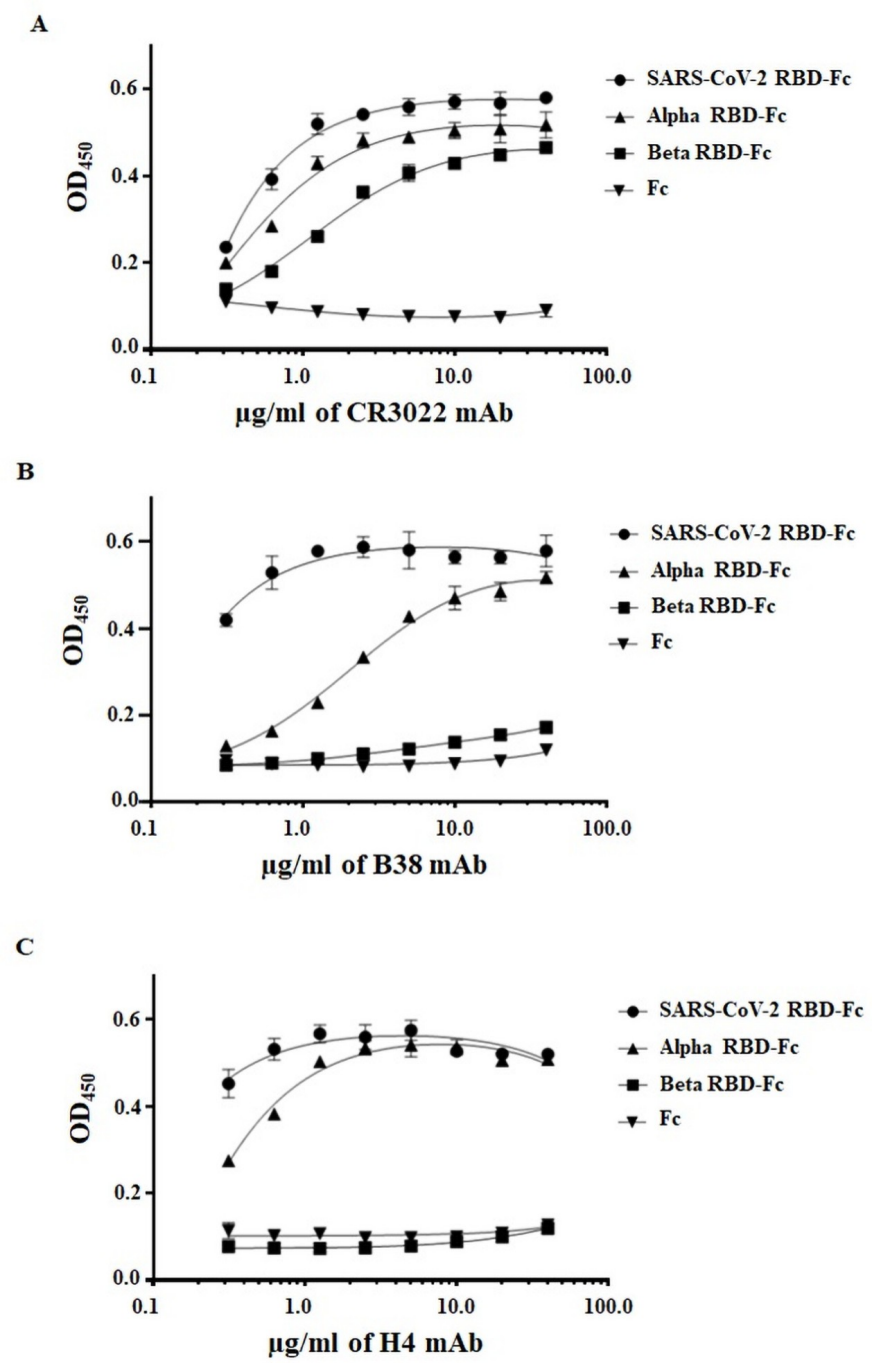
*In vitro* binding of purified plant-produced SARS-CoV-2 RBD-Fc and variants to mAbs against SARS-CoV-2 RBD. The binding affinity of plant-produced mAbs to plant-produced SARS-CoV-2 RBD-Fc and variants was determined by ELISA. The different concentration of anti-SARS-CoV mAb CR3022 (A) and anti-SARS-CoV-2 mAbs B38 (B), and H4 (C) were incubated on plates coated with plant-produced SARS-CoV-2 RBD-Fc, Alpha RBD-Fc, Beta RBD-Fc, and Fc proteins. The bound antibody was detected with HRP-conjugated goat anti-human Kappa. The data are the mean±SD of triplicates assay from each concentration.

The previous studies showed that the SARS-CoV-2 RBD can bind to anti-SARS-CoV mAb CR3022 [19] and anti-SARS-CoV-2 H4 and B38 mAbs [17]. To determine the effect of plant-produced RBD-Fc (SARS-CoV-2, Alpha, and Beta variant) on mAb binding, the purified plant-produced RBD-Fc of three strains were tested with mAb CR3022, B38, and H4. The plant-produced Fc protein was used as the negative control. The plant-produced SARS-CoV-2 RBD-Fc could bind to all anti-SARS-CoV mAb (CR3022) and anti-SARS-CoV-2 mAbs (B38, and H4) (Fig 5). The binding of plant-produced SARS-CoV-2 RBD-Fc to anti-SARS-CoV-2 mAbs was higher than the plant-produced Alpha RBD-Fc and Beta RBD-Fc variants. The plant-produced Alpha RBD-Fc binds to CR3022, B38 and H4 (Fig 5A–5C). However, its binding to B38 varied with antibody concentration (Fig 5B). In contrary, the plant-produced Beta RBD-Fc binds to CR3022 (Fig 5A), but not B38 and H4 (Fig 5B and 5C). These results showed that the mutation on RBD of SARS-CoV-2 of Beta RBD-Fc could affect mAb binding.

## Discussion

The COVID-19 pandemic caused by the novel SARS-CoV-2 has overwhelming drastic impacts on the human population, public health care system, and global economy. Recently, new genetic variants of SARS-CoV-2 have been identified. These variants contain diverse mutations which confers enhanced affinity for ACE2 [12] and provide competitive advantages to the virus in terms of infection, spread, and escape from neutralizing antibodies [2, 10, 11]. In addition, genetic variations seen across SARS-CoV-2 may significantly affect the efficacy of currently developed vaccines [23, 24]. Thus, a matter of intense interest over the SARS-CoV-2 variants continues to grow and necessitates active surveillance and investigation.

The new variants of SARS-CoV-2 have emerged recently and rapidly spread to many countries. The variants of concern, such as the lineage B.1.1.7 (20I/501Y.V1 or VOC 202012/01) and the lineage B.1.351 (20H/501Y.V2), were initially identified in United Kingdomand South Africa, respectively [6, 8]. For all variants, the mode of infection or viral entry into host cells starts with the interaction of S protein of SARS-CoV-2 with host cell receptor. In particular, the RBD found in the S protein binds to the human ACE2 cell receptor and then initiates viral attachment, membrane fusion and entry to cells [25, 26]. Hence, RBD of SARS-CoV-2 plays an important role in virus pathogenicity and is considered as one of the major antigenic targets for vaccine development. In comparison with the predominant SARS-CoV-2, the viral variants harbor multiple mutations in the RBD. For the virus lineage B.1.1.7 (Alpha), the amino acid mutations in RBD replaces asparagine with tyrosine at position 501 (N501Y), alanine to aspartic acid at position 570 (A570D), and aspartic acid to glycine at position 614 (D614G) [7]. The virus lineage B.1.351 (Beta) contains point substitutions in the RBD leads to amino acid change from asparagine to tyrosine at position 501 (N501Y), aspartic acid to glycine at position 614 (D614G), lysine to asparagine at position 417 (K417N), and glutamic acid to lysine at position 484 (E484K) [9]. These mutations in the SARS-CoV-2 RBD might confer differences in binding against the functional ACE2 receptor or neutralizing monoclonal antibodies, thus warrants further investigations. Hence, in this study, the RBD of the SARS-CoV-2, Alpha, and Beta variant was produced and its binding efficiency with the three reported mAbs CR3022, H4 and B38 were tested as a preliminary approach to evaluate the ability of mAbs to recognize emerging novel SARS-CoV-2 variants.

Many expression systems are available to produce recombinant vaccine antigens and mAbs. However, each system has their own advantages and limitations. Recently plants are considered as an effective platform to produce biopharmaceuticals especially during pandemics [25, 27–30]. In this study, the plant transient expression system was utilized to produce recombinant proteins due to its rapidity and scalability [31]. By transient expression, the recombinant proteins can be produced in less than 2 weeks after synthetic gene construct delivery [19, 32]. Geminiviral vector derived from the bean yellow dwarf virus was widely used for transient expression in plants as it produces very high levels of recombinant DNA for the protein of interest inside the leaf cells which in turn leads to high protein accumulation within 5 days after infiltration [18, 33]. Earlier reports showed proof-of-concept pilot-scale production of SARS-CoV-2 related proteins and mAbs in plants. Our group has already reported the production of SARS-CoV mAb CR3022 and SARS-CoV-2 mAbs H4 and B38 in plants [17, 19, 26, 34]. Here, we have used geminiviral vector for the production of SARS-CoV-2 RBD-Fc, Alpha RBD-Fc, and Beta RBD-Fc in *Nicotiana benthamiana*. The protein accumulation of approximately 20 μg/g leaf fresh weight was observed within 3 days post-infiltration.

The plant-produced RBD-Fc was purified and its ability to bind to cell receptor ACE2 was determined by ELISA. Our data showed that Alpha RBD-Fc protein strongly binds to human ACE2 compared to SARS-CoV-2 RBD-Fc and Beta RBD-Fc. The binding of Beta RBD-Fc to ACE2 was comparable with SARS-CoV-2 RBD-Fc. Our result was in consistent with the previous report in which D614G (Alpha and Beta) mutation enhances virus infection [35]. The point mutations N501Y (Alpha and Beta) and E484K (Beta) in the RBD provide strong binding between spike and ACE2 receptor in the novel variants [36, 37]. In contrast, the K417N (Beta) substitution in the RBD disrupts the binding of spike with ACE2 receptor [36].

**Fig 6:**
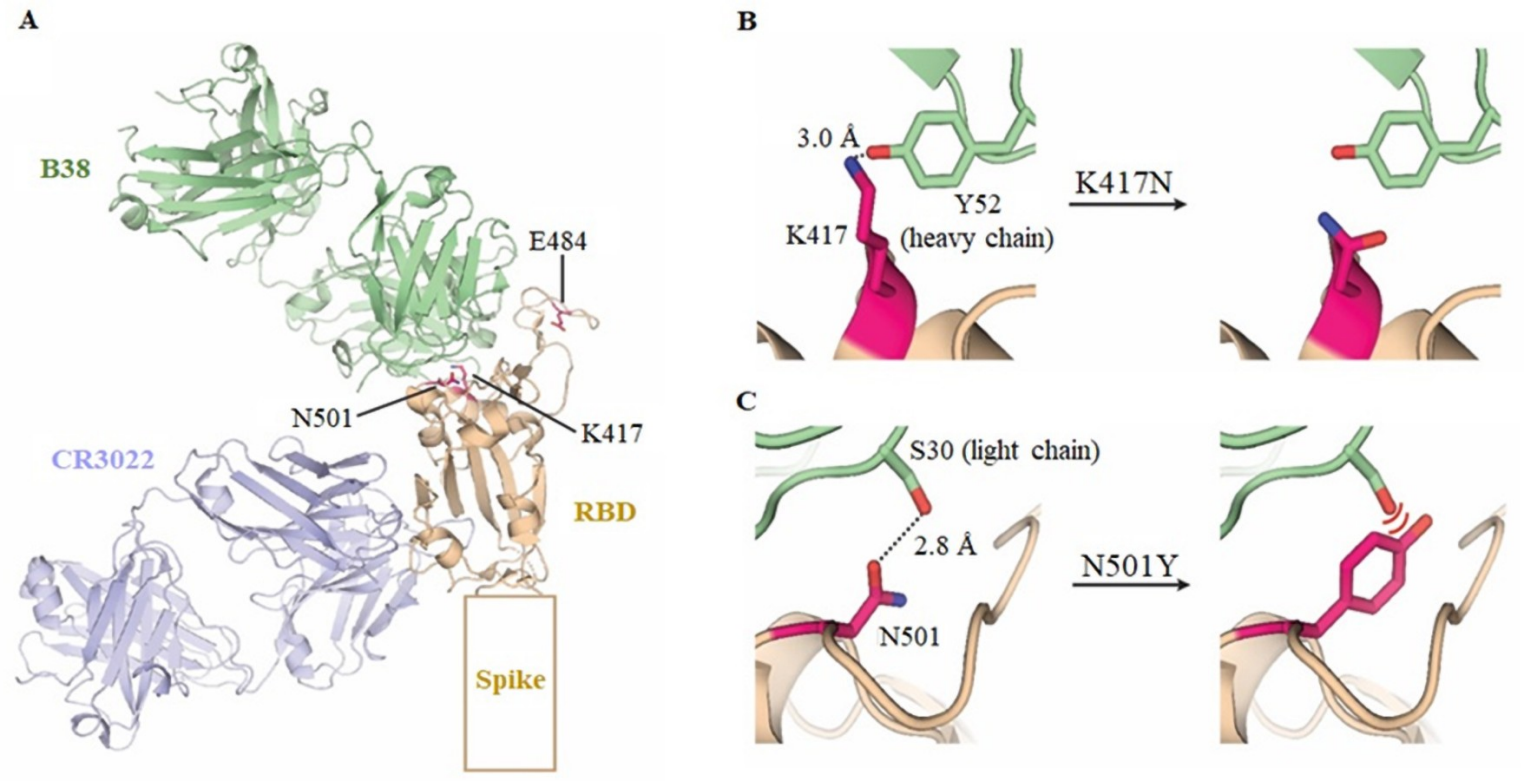
Structural analysis of the interactions between the SARS-CoV-2 RBD variants and mAb against SARS-CoV-2 RBD. (A) The overall structure of anti-SARS-CoV mAb CR3022 and anti-SARS-CoV-2 mAb B38 bound with SARS-CoV-2 RBD. (B) The potential mechanism of reduction in binding affinity due to the loss of an interaction in the K417N mutation in SARS-CoV-2 RBD. (C) The potential mechanism of reduction in binding affinity due to a steric clash from the N501Y mutation in the SARS-CoV-2 RBD.

In addition, the binding efficiency of the plant-produced RBD variants with mAbs were evaluated. The earlier reports from our group showed that the plant-produced anti-SARS-CoV mAb CR3022 [19] and SARS-CoV-2 mAbs B38, and H4 [17] can effectively bind the SARS-CoV-2 RBD protein. The impact of the mutations in the RBD of SARS-CoV-2 variants on the binding efficiency of three reported plant-produced mAbs CR3022, B38, and H4 was examined by ELISA. The results showed that the plant-produced SARS-CoV-2 RBD-Fc bind to three tested plant-produced mAbs. Whereas, plant-produced Alpha RBD-Fc variant showed similar binding affinity like plant-produced SARS-CoV-2 RBD-Fc against plant-produced CR3022 and H4 mAbs. However, the binding of Alpha RBD-Fc variant to plant-produced B38 mAb exhibited dose-dependent manner. Interestingly, the results of binding efficiency of Beta RBD-Fc with the mAbs showed that, it binds to the plant-produced CR3022 mAb but not with plant-produced B38 and H4 mAbs. Earlier studies reported that D614G (Alpha and Beta) mutation in the S protein enhances viral infectivity but it did not affect the binding of SARS-CoV-2 S protein to anti-SARS-CoV-2 sera [35]. In contrast, the E484K (Beta) mutation is of great concern, since it enhances binding affinity with ACE2 and reduces the binding interactions with neutralizing antibodies [37] and polyclonal serum antibodies [38] compromising the neutralization. Similar results were also reported in which the sensitivity to antibody neutralization varies between Alpha and Beta variant, however Beta variant was reported to be less sensitive to neutralizing antibodies and could potentially be more problematic than the Alpha variant [39]. Based on our structural analysis, the mutations in the SARS-CoV-2 RBD did not affect the binding efficacy of the CR3022 mAb, whereas mutations at N501Y (Alpha and Beta) and K417N (Beta) have the effect on the binding of SARS-CoV-2 RBD to anti-SARS-CoV-2 mAb B38 and H4 (Fig 6). Future work with sera from the diverse group including the recovered patients and vaccine recipients will help to demonstrate the differential binding of plant-produced RBD of variant strains and the role of variants in vaccine efficacy.

To rationalize the differential binding affinity of SARS-CoV-2 RBD to mAbs, the structures of SARS-CoV-2 RBD in complex with the Fab fragments of the CR3022 [20] and B38 [21] antibodies were examined (Fig 6A). The CR3022 mAb bound in the location remote from K417, E484, and N501 residues of the RBD. Therefore, mutation in these locations should not drastically affect the binding affinity of CR3022 to RBD. Indeed, our ELISA results show the CR3022 mAb could still bind the SARS-CoV-2 RBD. The B38 mAb bound in the region of RBD where mutations are present and is expected to be affected by the mutations. The Beta RBD contains the K417N and N501Y mutations that are at the B38 mAb binding interface. K417 normally forms a hydrogen bond with Y52 of the B38 mAb heavy chain (Fig 6B). The K417N mutation introduces asparagine with the shorter side chain that could no longer form a hydrogen bond with Y52. The N501 in SARS-CoV-2 RBD forms a hydrogen bond with S30 of the B38 mAb light chain (Fig 6C). The N501Y mutation converted the small asparagine into the bulky tyrosine that resulted in a steric clash with the B38 mAb. The combination of both the K417N and N501Y mutations should renders the B38 mAb a weak binder of Beta RBD, which agrees with our ELISA results. The Alpha RBD only contained the N501Y in the B38 mAb-binding region. Therefore, a reduction in the binding affinity towards B38 mAb is expected, but to a lesser degree compared to the Beta RBD variant. Our ELISA results also corroborated with the structural analysis. Our structural analysis not only confirms the experimental observation, but also highlight to functional reproducibility of RBD produced in plant-based system compared to other expression systems.

To date, no three-dimensional structural information about the H4 antibody binding location on the RBD is available. It is known that the H4 mAb could compete with the B38 antibody. The epitopes are likely different but with partial overlap [21]. From our ELISA results, the H4 mAb could still bind the Alpha RBD. Because the N501Y is the only mutation in the Alpha strain has in the RBD, our data suggests that the epitope recognized by the H4 antibody does not involve N501. The H4 mAb could not bind the Beta RBD at all. It is possible that the H4 epitope could involve both K417 and E484. More mutagenesis experiments should be done to determine whether the single K417N or E484K mutation, or both the K417N and E484K are required to disrupt the H4 mAb binding.

In conclusion, our results demonstrate that RBD of SARS-CoV-2 and variants can be produced in plant expression system and the preliminary results on the binding efficiency of plant-produced RBDs with three tested mAbs confirmed that the binding efficacy of Alpha RBD with B38 mAb varied depending on the concentration and that the Beta RBD has less binding affinity with the studied antibodies. The findings implied that these Alpha and Beta variants could be possible threat for previously immune populations. However, detailed studies with the range of neutralizing antibodies are required to confirm these findings. The plant-produced RBD variants could mimic the structure of mutant SARS-CoV-2 RBD and possibly provide information on the effective vaccine design for mutant strains.

## Supporting information

S1 File (DOCX)

## References

[1] WHO. COVID-19 status report 2021. https://covid19.who.int/.

[2] Burki T. Understanding variants of SARS-CoV-2. Lancet (London, England). 2021;397(10273):462. doi: 10.1016/S0140-6736(21)00298-1 33549181 PMC7906644

[3] Public-Health-England. Investigation of novel SARS-CoV-2 variant: Variant of Concern 202012/01. 2020.

[4] Tegally H, Wilkinson E, Giovanetti M, Iranzadeh A, Fonseca V, Giandhari J, et al. Emergence and rapid spread of a new severe acute respiratory syndrome-related coronavirus 2 (SARS-CoV-2) lineage with multiple spike mutations in South Africa. medRxiv. 2020:2020.12.21.20248640.

[5] WHO. Tracking SARS-CoV-2 variants 2021. https://www.who.int/en/activities/tracking-SARS-CoV-2-variants/.

[6] Davies NG, Abbott S, Barnard RC, Jarvis CI, Kucharski AJ, Munday JD, et al. Estimated transmissibility and impact of SARS-CoV-2 lineage B.1.1.7 in England. Science. 2021:eabg3055. doi: 10.1126/science.abg3055 33658326 PMC8128288

[7] Gu H, Chen Q, Yang G, He L, Fan H, Deng Y-Q, et al. Adaptation of SARS-CoV-2 in BALB/c mice for testing vaccine efficacy. Science. 2020;369(6511):1603. doi: 10.1126/science.abc4730 32732280 PMC7574913

[8] Giandhari J, Pillay S, Wilkinson E, Tegally H, Sinayskiy I, Schuld M, et al. Early transmission of SARS-CoV-2 in South Africa: An epidemiological and phylogenetic report. International journal of infectious diseases: IJID: official publication of the International Society for Infectious Diseases. 2021;103:234–41. 33189939 10.1016/j.ijid.2020.11.128 PMC7658561

[9] Tegally H, Wilkinson E, Giovanetti M, Iranzadeh A, Fonseca V, Giandhari J, et al. Emergence of a SARS-CoV-2 variant of concern with mutations in spike glycoprotein. Nature. 2021.

[10] Greaney AJ, Starr TN, Gilchuk P, Zost SJ, Binshtein E, Loes AN, et al. Complete Mapping of Mutations to the SARS-CoV-2 Spike Receptor-Binding Domain that Escape Antibody Recognition. Cell Host & Microbe. 2021;29(1):44–57.e9. doi: 10.1016/j.chom.2020.11.007 33259788 PMC7676316

[11] Pearson CAB, Timothy W Russell, Nicholas Davies, Adam J Kucharski, CMMID-COVID-19-working-group, Edmunds WJ, et al. Estimates of severity and transmissibility of novel SARS-CoV-2 variant 501Y.V2 in South Africa. 2021.

[12] Starr TN, Greaney AJ, Hilton SK, Ellis D, Crawford KHD, Dingens AS, et al. Deep Mutational Scanning of SARS-CoV-2 Receptor Binding Domain Reveals Constraints on Folding and ACE2 Binding. Cell. 2020;182(5):1295–310.e20. doi: 10.1016/j.cell.2020.08.012 32841599 PMC7418704

[13] Wang Z, Schmidt F, Weisblum Y, Muecksch F, Barnes CO, Finkin S, et al. mRNA vaccine-elicited antibodies to SARS-CoV-2 and circulating variants. Nature. 2021. 10.1038/s41586-021-03324-6 PMC8503938 33567448

[14] Andreano E, Piccini G, Licastro D, Casalino L, Johnson NV, Paciello I, et al. SARS-CoV-2 escape from a highly neutralizing COVID-19 convalescent plasma. bioRxiv. 2020:2020.12.28.424451. doi: 10.1101/2020.12.28.424451 34417349 PMC8433494

[15] Wibmer CK, Ayres F, Hermanus T, Madzivhandila M, Kgagudi P, Oosthuysen B, et al. SARS-CoV-2 501Y.V2 escapes neutralization by South African COVID-19 donor plasma. Nature Medicine. 2021. 10.1038/s41591-021-01285-x 33654292

[16] Siriwattananon K, Manopwisedjaroen S, Shanmugaraj B, Rattanapisit K, Phumiamorn S, Sapsutthipas S, et al. Plant-Produced Receptor-Binding Domain of SARS-CoV-2 Elicits Potent Neutralizing Responses in Mice and Non-human Primates. Frontiers in plant science. 2021;12:682953-. doi: 10.3389/fpls.2021.682953 34054909 PMC8158422

[17] Shanmugaraj B, Rattanapisit K, Manopwisedjaroen S, Thitithanyanont A, Phoolcharoen W. Monoclonal Antibodies B38 and H4 Produced in Nicotiana benthamiana Neutralize SARS-CoV-2 in vitro. Frontiers in plant science. 2020;11:589995. doi: 10.3389/fpls.2020.589995 33329653 PMC7728718

[18] Chen Q, He J, Phoolcharoen W, Mason HS. Geminiviral vectors based on bean yellow dwarf virus for production of vaccine antigens and monoclonal antibodies in plants. Human vaccines. 2011;7(3):331–8. doi: 10.4161/hv.7.3.14262 21358270 PMC3166492

[19] Rattanapisit K, Shanmugaraj B, Manopwisedjaroen S, Purwono PB, Siriwattananon K, Khorattanakulchai N, et al. Rapid production of SARS-CoV-2 receptor binding domain (RBD) and spike specific monoclonal antibody CR3022 in Nicotiana benthamiana. Scientific Reports. 2020;10(1):17698. doi: 10.1038/s41598-020-74904-1 33077899 PMC7573609

[20] Yuan M, Wu NC, Zhu X, Lee CD, So RTY, Lv H, et al. A highly conserved cryptic epitope in the receptor binding domains of SARS-CoV-2 and SARS-CoV. Science. 2020;368(6491):630–3. doi: 10.1126/science.abb7269 32245784 PMC7164391

[21] Wu Y, Wang F, Shen C, Peng W, Li D, Zhao C, et al. A noncompeting pair of human neutralizing antibodies block COVID-19 virus binding to its receptor ACE2. Science. 2020;368(6496):1274–8. doi: 10.1126/science.abc2241 32404477 PMC7223722

[22] Hoffmann M, Kleine-Weber H, Schroeder S, Krüger N, Herrler T, Erichsen S, et al. SARS-CoV-2 Cell Entry Depends on ACE2 and TMPRSS2 and Is Blocked by a Clinically Proven Protease Inhibitor. Cell. 2020;181(2):271–80.e8. doi: 10.1016/j.cell.2020.02.052 32142651 PMC7102627

[23] Dearlove B, Lewitus E, Bai H, Li Y, Reeves DB, Joyce MG, et al. A SARS-CoV-2 vaccine candidate would likely match all currently circulating variants. Proceedings of the National Academy of Sciences. 2020;117(38):23652. doi: 10.1073/pnas.2008281117 32868447 PMC7519301

[24] Eguia R, Crawford KHD, Stevens-Ayers T, Kelnhofer-Millevolte L, Greninger AL, Englund JA, et al. A human coronavirus evolves antigenically to escape antibody immunity. bioRxiv. 2020:2020.12.17.423313. 10.1371/journal.ppat.1009453 PMC8031418 33831132

[25] Shanmugaraj B, Malla A, Phoolcharoen W. Emergence of Novel Coronavirus 2019-nCoV: Need for Rapid Vaccine and Biologics Development. Pathogens (Basel, Switzerland). 2020;9(2). doi: 10.3390/pathogens9020148 32098302 PMC7168632

[26] Siriwattananon K, Manopwisedjaroen S, Kanjanasirirat P, Budi Purwono P, Rattanapisit K, Shanmugaraj B, et al. Development of Plant-Produced Recombinant ACE2-Fc Fusion Protein as a Potential Therapeutic Agent Against SARS-CoV-2. Frontiers in plant science. 2020;11:604663. doi: 10.3389/fpls.2020.604663 33584747 PMC7874119

[27] Capell T, Twyman RM, Armario-Najera V, Ma JK, Schillberg S, Christou P. Potential Applications of Plant Biotechnology against SARS-CoV-2. Trends in plant science. 2020;25(7):635–43. doi: 10.1016/j.tplants.2020.04.009 32371057 PMC7181989

[28] Sainsbury F. Innovation in plant-based transient protein expression for infectious disease prevention and preparedness. Current opinion in biotechnology. 2020;61:110–5. doi: 10.1016/j.copbio.2019.11.002 31816585 PMC7127347

[29] Shanmugaraj B, Siriwattananon K, Wangkanont K, Phoolcharoen W. Perspectives on monoclonal antibody therapy as potential therapeutic intervention for Coronavirus disease-19 (COVID-19). Asian Pacific journal of allergy and immunology. 2020;38(1):10–8. doi: 10.12932/AP-200220-0773 32134278

[30] Tusé D, Nandi S, McDonald KA, Buyel JF. The Emergency Response Capacity of Plant-Based Biopharmaceutical Manufacturing-What It Is and What It Could Be. 2020;11(1573). 10.3389/fpls.2020.594019 PMC7606873 33193552

[31] Shanmugaraj B, CJ IB, Phoolcharoen W. Plant Molecular Farming: A Viable Platform for Recombinant Biopharmaceutical Production. Plants (Basel, Switzerland). 2020;9(7). doi: 10.3390/plants9070842 32635427 PMC7411908

[32] Lomonossoff GP D’Aoust MA. Plant-produced biopharmaceuticals: A case of technical developments driving clinical deployment. Science. 2016;353(6305):1237–40. doi: 10.1126/science.aaf6638 27634524

[33] Diamos AG, Mason HS. Modifying the Replication of Geminiviral Vectors Reduces Cell Death and Enhances Expression of Biopharmaceutical Proteins in Nicotiana benthamiana Leaves. Frontiers in plant science. 2018;9:1974. doi: 10.3389/fpls.2018.01974 30687368 PMC6333858

[34] Diego-Martin B, González B, Vazquez-Vilar M, Selma S, Mateos-Fernández R, Gianoglio S, et al. Pilot Production of SARS-CoV-2 Related Proteins in Plants: A Proof of Concept for Rapid Repurposing of Indoor Farms Into Biomanufacturing Facilities. 2020;11(2101). doi: 10.3389/fpls.2020.612781 33424908 PMC7785703

[35] Ozono S, Zhang Y, Ode H, Sano K, Tan TS, Imai K, et al. SARS-CoV-2 D614G spike mutation increases entry efficiency with enhanced ACE2-binding affinity. Nature Communications. 2021;12(1):848. doi: 10.1038/s41467-021-21118-2 33558493 PMC7870668

[36] Padilla-Sanchez V. SARS-CoV-2 Structural Analysis of Receptor Binding Domain New Variants from United Kingdom and South Africa. Research Ideas and Outcomes. 2021;7:e62936.

[37] Wang WB, Liang Y, Jin YQ, Zhang J, Su JG, Li QM. E484K mutation in SARS-CoV-2 RBD enhances binding affinity with hACE2 but reduces interactions with neutralizing antibodies and nanobodies: Binding free energy calculation studies. bioRxiv. 2021:2021.02.17.431566. 10.1016/j.jmgm.2021.108035 PMC8447841 34562851

[38] Greaney AJ, Loes AN, Crawford KHD, Starr TN, Malone KD, Chu HY, et al. Comprehensive mapping of mutations to the SARS-CoV-2 receptor-binding domain that affect recognition by polyclonal human serum antibodies. bioRxiv. 2021:2020.12.31.425021. 10.1016/j.chom.2021.02.003 PMC7869748 33592168

[39] Planas D, Bruel T, Grzelak L, Guivel-Benhassine F, Staropoli I, Porrot F, et al. Sensitivity of infectious SARS-CoV-2 B.1.1.7 and B.1.351 variants to neutralizing antibodies. Nat Med. 2021;27(5):917–24. doi: 10.1038/s41591-021-01318-5 33772244

