# Supplementary material for: Plant-produced SARS-CoV-2 receptor binding domain (RBD) variants showed differential binding efficiency with anti-spike specific monoclonal antibodies
Source: PLoS One. 2021 Aug 11;16(8):e0253574. doi: 10.1371/journal.pone.0253574 (PMC8357147; doi:10.1371/journal.pone.0253574)
Supplement: S1 File — (DOCX) [file pone.0253574.s001.docx]

**Plant-Produced SARS-CoV-2 Receptor Binding Domain (RBD) Variants Showed Differential Binding Efficiency with Anti-Spike Specific Monoclonal Antibodies**

Kaewta Rattanapisit^1^, Christine Joy I. Bulaon^2,3^, Narach Khorattanakulchai^2,3^, Balamurugan Shanmugaraj^1^, Kittikhun Wangkanont^4,5^, Waranyoo Phoolcharoen^2,3*^

**Supplementary figure**

Figure 2 (Uncropped)


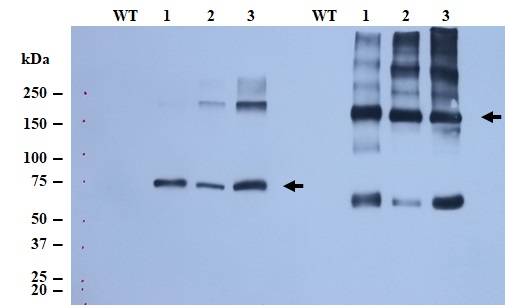


**Figure 2.** Transient expression of SARS-CoV-2 RBD-Fc and variants in *N. benthamiana*. Agroinfiltrated *N. benthamiana* leaves were extracted and protein expression was assessed by western blotting using HRP-conjugated goat anti-human IgG Fc specific antibody under reducing condition (Left) and non-reducing condition (Right). Lane WT: crude extract of wild type *N. benthamiana*; Lane 1: crude extract of *N. benthamiana* agroinfiltrated with pBY2eK-SARS-CoV-2 RBD-Fc; Lane 2: crude extract of *N. benthamiana* agroinfiltrated with pBY2eK-Beta RBD-Fc; Lane 3: Lane 1: crude extract of *N. benthamiana* agroinfiltrated with pBY2eK-Alpha RBD-Fc. The arrow indicated the expected band. The experiment was performed in three times repeat.

Figure 3 (Uncropped)


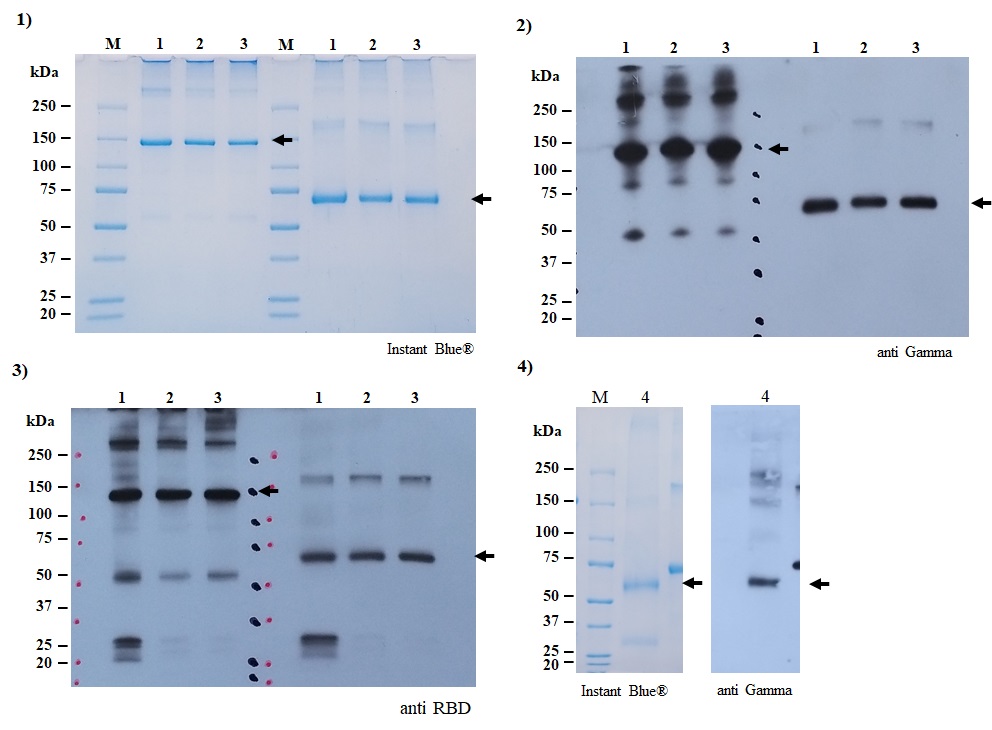


**Figure 3.** SDS-PAGE and western blot analysis of purified plant-produced SARS-CoV-2 RBD-Fc, variants, and Fc. Purified plant-produced SARS-CoV-2 RBD-Fc, Alpha RBD-Fc, and Beta RBD-Fc variants were evaluated by SDS-PAGE and western blot under reducing condition (Right of 1, 2, and 3) and non-reducing condition (Left of 1, 2, and 3). In addition, purified plant-produced Fc was assessed by SDS-PAGE and western blot under nonreducing condition (4). The proteins were separated by 4-15% SDS PAGE and stained with InstantBlue® (1). For western blot, the proteins were transferred onto nitrocellulose membrane and probed with HRP-conjugated goat anti-human IgG (2) or rabbit anti-SARS-CoV-2 spike protein (RBD) mAb (3). Lane 1: purified plant-produced SARS-CoV-2 RBD-Fc; Lane 2: purified plant-produced Beta RBD-Fc protein; Lane 3: purified plant-produced Alpha RBD-Fc protein; Lane 4: purified plant-produced Fc protein. The arrow indicated the expected band.
